# Electroacupuncture at GV20 and ST36 Exerts Neuroprotective Effects via the EPO-Mediated JAK2/STAT3 Pathway in Cerebral Ischemic Rats

**DOI:** 10.1155/2017/6027421

**Published:** 2017-08-07

**Authors:** Hong Xu, Ya-min Zhang, Hua Sun, Su-hui Chen, Ying-kui Si

**Affiliations:** Department of Traditional Chinese Medicine, Peking Union Medical College Hospital (PUMCH), Peking Union Medical College (PUMC), Chinese Academy of Medical Sciences, Beijing 100730, China

## Abstract

**Background:**

While electroacupuncture (EA) in cerebral ischemia has been used to promote functional recovery, the underlying mechanism of its protective effect remains poorly understood.

**Objective:**

We investigated the effects of EA stimulation at GV20 and ST36 to observe the changes in erythropoietin- (EPO-) mediated Janus family tyrosine kinases 2 (JAK2) signal transducers and activators of the transcription 3 (STAT3) cell pathway.

**Methods:**

Thirty-six specific pathogen-free Sprague-Dawley (SD) male rats were randomly assigned to three groups: the sham-operated group (S group), the middle cerebral artery occlusion (MCAO) group (M group), and the EA group. Neurological deficits were assessed through the Ludmila Belayev 12-score test and 2,3,5-triphenyltetrazolium chloride (TTC) staining was shown. The protein and mRNA expression levels of EPO, the EPO receptor (EpoR), p-JAK2, JAK2, p-STAT3, and STAT3 were examined to explore the EA effect on rats with cerebral ischemic reperfusion injury (CIRI).

**Results:**

EA significantly decreased infarct size and improved neurological function. Furthermore, target EPO, EpoR, JAK2, and STAT3 mRNA and protein levels significantly increased in the EA group.

**Conclusions:**

EA exerts a neuroprotective effect, possibly via the regulation of the EPO-mediated JAK2/STAT3 cell pathway and downstream apoptotic pathways in a rat CIRI model.

## 1. Introduction

As a primary cause of paralysis and death around the world, ischemic stroke and the accompanying reperfusion injury are widely known for such complications as hemiplegia, coma, and even death. Although new and advanced tools and medications are available for earlier diagnosis and treatment, such as magnetic resonance imaging (MRI) technology [1, 2], high morbidity and mortality still constitute the most disturbing prognosis of this vascular disease. Many pathological changes happen during the obstinate illness, including cell death (necrosis and apoptosis), angiogenesis, inflammation, and cerebral edema [3–5]. Novel therapeutic agents and targets that are safe, efficacious treatment options are needed for patients with this complex disease because of the limits of the clinical use of clot-lysing drugs.

As a hematopoietic factor that is normally used as a medicine for the treatment of anemia, the 165 kDa secreted glycoprotein EPO has a neuroprotective function in the central nervous system (CNS). The EPO of the CNS may come from two pathways: one part comes from peripheral organs and the other part is expressed by CNS cells. As a pleiotropic factor, EPO can be induced in many central nervous cells, such as neurons, astrocytes, and endothelial cells, in rats with cerebral ischemic reperfusion injuries [6]. EPO can serve as both a cytoprotective and a neuroprotective factor in CNS injury. One of the verified functions of EPO in the CNS was a reduction in infarct size in acute ischemic stroke in a clinical study [7]. The effect of preserving brain structure and function was also induced by EPO treatment in animals undergoing brain ischemia [8]. Many mechanisms are involved in the function of EPO after brain ischemia or CIRI. EPO, which can be activated by hypoxia, was demonstrated to be important in the protection of neurons from autoimmune-mediated CNS inflammation [9], preventing neuronal apoptosis [10] and inducing intrinsic neuron axon regeneration [11] in animal models. Proteins and molecules, such as JAK2 and STAT3, were correspondingly changed by EPO in models of hypoxic/ischemic insult.

In CNS diseases, such as brain ischemia, the EpoR connects EPO to JAK2. After binding to the EpoR, EPO is activated. Next, the EpoR, which belongs to class I cytokine receptor family, is dimerized and binds to JAK2. The substrates of JAK2 can phosphorylate STAT3, and the JAK2/STAT3 complex translocates to the nucleus. One research study verified that EPO administration after CIRI could activate JAK2 in a hypoxic-ischemic neonatal model [12]. The EPO-mediated-JAK2/STAT3 cell signaling pathway can be concluded to influence the expression of many genes related to the recovery of CNS diseases.

The JAK/STAT pathway is highly important in cell signal transduction. Four types of JAK (JAK1, JAK2, JAK3, and Tyk2) and seven types of STAT (STAT1, STAT2, STAT3, STAT4, STAT5a, STAT5b, and STAT6) proteins exist. Of all of these subtypes, JAK2 and STAT3 have been studied the most by researchers. The JAK family of kinases has been shown to bind to such growth factors as EPO and interleukins. These kinases can couple with the receptor of the STAT family of proteins. The role of JAK2/STAT3-mediated apoptosis following CIRI has been demonstrated by many researchers [13].

EA stimulation at the acupoints Baihui (GV20) and Zusanli (ST36) improves the recovery of neurological function following many CNS diseases, such as subarachnoid hemorrhage[14], but its mechanism remains incompletely understood. Our former research confirmed that acupuncture treatment could reduce brain damage and related behavioral deficits through many mechanisms, such as the reduction of brain edema and the attenuation of inflammatory damage [15, 16]. In this study, we hypothesized that the neuroprotective effect of the EPO related-JAK2/STAT3 cell pathway was activated by acupointing at Baihui (GV20) and Zusanli (ST36) in a focal cerebral ischemic model using rats. We also hypothesized that acupointing at GV20 and ST36 could activate the STAT3-mediated apoptosis pathway.

## 2. Materials and Methods

### 2.1. Animals

A total of 38 pathogen-free, adult male SD rats weighing 230–250 g (Peking Union Medical College Hospital) were used in this study. Two rats died during the surgery. The animals were housed in cages in a room maintained at 22 ± 2°C, 60%  ±  5% humidity, and a 12 h light/dark cycle with free access to rodent chow and tap water. The study protocol and all subsequent amendments were approved by the Ethics Committee of Peking Union Medical College Hospital and Chinese Academy of Medical Sciences.

### 2.2. Transient Focal Cerebral Ischemia-Reperfusion Model and Surgical Procedures

Focal cerebral ischemic rat models were induced by MCAO as previously described by Longa et al. [17]. The rats were anesthetized with an intraperitoneal injection of 10% chloral hydrate (0.35 mL/100 g). After a midline neck incision, the right carotid bifurcation was fully exposed, and the external carotid artery (ECA) was ligated distal to the bifurcation with a nylon suture. A poly-L-lysine-coated 4-0 suture was subsequently inserted into the internal carotid artery (ICA) through the ECA. The suture was gently advanced 18–20 mm further until mild resistance was felt, and it effectively occluded the trunk of the middle cerebral artery. After 2 h of MCAO, reperfusion was initiated by removing the silk sutures to restore MCA reperfusion.

### 2.3. Experimental Protocol

The rats were randomly assigned to the following groups: the S group, the M group, and the EA group (12 rats in each group). The M and EA groups underwent MCAO through the blockage of the cerebral blood flow of the right MCA for 2 h, and reperfusion was allowed for 24 h. The rats in group S received the same surgical procedures as those in group M but without blockage of the MCA by nylon monofilament. EA group rats were given two EA treatments. The first treatment was conducted after MCAO for 2 h, and the second was conducted 2 h before euthanasia. In the EA group, a 20-minute EA with the application of a pulsating electrical current to the acupuncture needles was operated by an acupunctoscope device to stimulate the acupoints (Changzhou Wujin Great Wall Medical Instrument Co., Ltd., Chang Zhou, China). The needles were inserted into the paralysis limbs at the acupuncture points (left ST36) and GV20. The rats accepted EA treatment at a depth of 10 mm in GV20 (located in the frontal lobe of the anterior precentral sulcus) and at approximately 10 mm in ST36 (anatomically located near the knee joint of the hind limb 2 mm lateral to the anterior tubercle of the tibia). The EA parameter was set as frequency 2–100 Hz and intensity 2 mA, which was below the threshold of muscle contraction and matched the endurance of the rats.

### 2.4. Neurological Score Assessment

Neurological function was tested in all 36 rats to quantify neurological function using the Ludmila Belayev 12-score test [18]. A blinded assessment of the neurological examination grading outcome was described after the EA treatment. The two parts of the examination included (graded on a scale of 0 to 12) postural-reflex and forelimb-placing tests. The postural-reflex text was applied to examine upper body posture by suspending the tail of the rats (scale: 0 to 2) and the forelimb-placing test was used to quantify the damage to sensorimotor function during tests of contact, including chin, proprioception, and visual placing reactions (scale: 0 to 10).

### 2.5. TTC Staining

After the intraperitoneal injection of 10% chloral hydrate, TTC staining was used to evaluate the infarct volume. Rat brain tissues were immediately collected and placed in −20°C refrigerator for 10 min. Five 2 mm thick coronal sections were taken serially using a special brain matrix for rats. The brain slices were put into a solution of 1% TTC (Sigma, USA) in phosphate buffer saline (PBS) in an oven at 37°C for 30 min before being transferred into 4% paraformaldehyde for 1 h and later photographed. The percentage of infarct volume was calculated according to the following formula: [(VC − VL)/VC] × 100%, where “VC” is the volume of the control hemisphere and “VL” is the noninfarcted tissue in the lesioned ipsilateral hemisphere[19]. Then the data were analyzed by an observer with no prior knowledge of the experiment.

### 2.6. Histopathological and Immunostaining

For EPO, EpoR, JAK2, and STAT3 immunostaining, slices (*n* = 6 for each group) were pretreated with Tris-Na-Blocking (TNB) blocking buffer (PerkinElmer, USA) containing 0.1 M Tris-HCl, pH 7.5, 0.15 M NaCl, and 0.5% blocking reagent before incubation with a primary antibody. Free-floating sections were immunolabeled with the following rabbit polyclonal antibodies from Santa Cruz Biotechnology (USA) overnight at 4°C: anti-EPO (Epo; 1 : 50); anti-EpoR (1 : 50); anti-JAK2 (1 : 100); and anti-STAT3 (1 : 100). Then, the PV-6001 Polink-1 HRP DAB Detection System, one-step polymer detection system, for mouse, rabbit, and rat antibodies was used at 37°C for 1 h according to manufacturer's instructions (GBI, Inc., USA). The slices were visualized by incubating with 0.5% diaminobenzidine (DAB). Finally, the number of immune positive cells in the penumbra area was calculated under a light microscope.

### 2.7. Immunofluorescence Staining

Double immunofluorescence staining was performed with primary antibodies against JAK2 (rabbit polyclonal, 1 : 100; Santa Cruz Biotechnology, USA) and GFAP (rabbit monoclonal, 1 : 200; Abcam, UK), JAK2 and NeuN (rabbit monoclonal, 1 : 200; Abcam, UK), and TUNEL (In situ cell death detection kit, Roche, USA) and EPO (rabbit polyclonal, 1 : 50; Santa Cruz Biotechnology, USA). Secondary antibodies were conjugated to FITC, Cy5, and Cy3, and the nuclei were stained with 4′,6-diamidino-2-phenylindole phenylindole (TSA Biotin Systems NEL700A001KT, PerkinElmer, USA). The TUNEL reaction and immunofluorescence staining were applied to fixed paraffin-embedded specimens according to the manufacturer's instructions. Immunostained slides were imaged by confocal microscopy (Olympus, Japan). Colocalization of JAK2 with GFAP, JAK2 with NeuN, and TUNEL with EPO was estimated using Pearson's correlation coefficient (PCC) [20].

### 2.8. Western Blot Analysis

The rats' brain tissues (*n* = 3) were lysed with an F60 Sonic Dismembrator (Fisher Scientific, USA) in radioimmunoprecipitation assay (RIPA) buffer (50 mM Tris-HCl, pH 7.5, 150 mM NaCl, 1% Triton X- 100, 1% sodium deoxycholate, Thermo) containing a protease inhibitor cocktail (Sigma, USA) with 1 mM DTT, 0.1 mM PMSF, 10 mM NaF, 1 mM Na_2_VO_3_, and 10 mM glycerophosphate. The tissues were later centrifuged in a microcentrifuge for 15 min at maximum speed. The soluble tissue lysates were transferred into new tubes. Equivalent amounts of lysates (20 *μ*g) were separated by SDS-PAGE, transferred to nitrocellulose, and subsequently probed by immunoblotting using standard procedures. Primary rabbit antibodies against EPO (1 : 200), EpoR (1 : 200), JAK2 (1 : 300), and STAT3 (1 : 300) and goat antibodies against p-JAK2 (1 : 300) were obtained from Santa Cruz. Secondary antibodies conjugated to horseradish peroxidase, including anti-rabbit IgG and anti-goat IgG, were obtained from Jackson and Luminol reagents from Millipore. Chemiluminescent signals within the linear range of detection were quantified using Image analysis software (Labwork 4.6). The expression ratios were normalized according to glyceraldehyde-3-phosphate dehydrogenase (GAPDH) levels.

### 2.9. Quantitative Real-Time Polymerase Chain Reaction (qPCR) Analysis

Expression levels of mRNA for EPO, EpoR, JAK2, and STAT3 were determined using qPCR with the Bio-Rad CFX96 Detection System (Applied Biosystems, USA) using the Plexor One-Step qPCR System (Promega A4021, USA) (*n* = 3 rats per group). For EPO, EpoR, JAK2, and STAT3, the following primers were used: EPO forward, 5′-GAATGAAGGTGGAAGAACAGGC-3′, and reverse, 5′-GCACCCGAAGCAGTGAAGTG-3′; EpoR forward, 5′-CTCTCAGTCTCGTCCTCATCTCAC-3′, and reverse, 5-GGCTACTTGGGCTCCACCA-3′; JAK2 forward, 5′-CAGATTCCGCAGGTTCATTC-3′ and reverse, 5′-CTTGTGGACGGTCACAACTCTAC-3′; STAT3 forward, 5′-GGAAAAGGACATCAGTGGCAAG-3′, and reverse, 5′-CGGCAGGTCAATGGTATTGC-3′. The relative quantification of the mRNA level was determined using the 2^−ΔΔCt^ method. The cDNA were amplified with primers for GAPDH (as an internal control) and the following GAPDH primers were used: forward, 5′-CACAGCAAGTTCAACGGCACAG-3′, and reverse, 5′-GACGCCAGTAGACTCCACGACA-3′.

### 2.10. Statistical Analysis

Mean values for each group were analyzed using a one-way ANOVA, and significant results were assessed with the LSD *t*-test. Statistical significance was assumed in all cases if *P* < 0.05.

## 3. Results

### 3.1. Administration of EA Improved Neurological Outcomes 2 h and 24 h after MCAO

**Figure 1:**
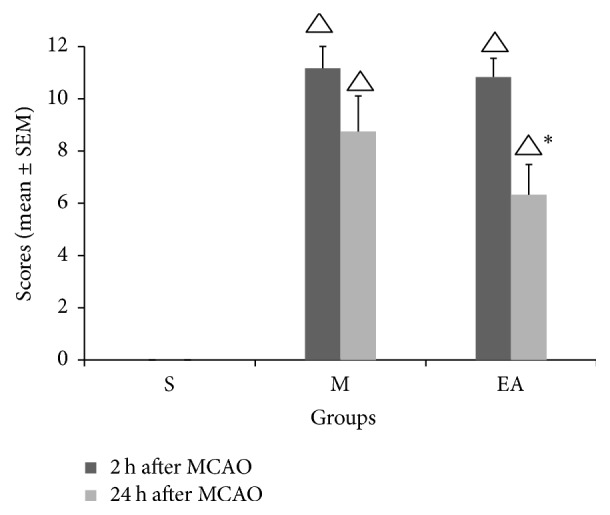
EA treatment improved the neurological outcomes. The scores of the Ludmila Belayev 12-score test were analyzed and are displayed. The animals in the S group showed the lowest scores, representing the best behavioral evaluation. The EA-treated rats had lower scores compared with the M group rats 24 h after MCAO, and the difference was significant. However, the difference between the two groups at 2 h was not significant. ^△^*P* < 0.05 compared to the S group, ^*∗*^*P* < 0.05 compared to the MCAO group. The graph shows the estimates for the scores (mean ± SEM).

After CIRI, we next tested whether the positive effect of EA treatment could be translated into functional improvement. To investigate the impact of EA stimulation on neurological outcomes, the Ludmila Belayev 12-score test was performed twice after EA treatment (2 h after MCAO and 24 h after MCAO). As illustrated in Figure 1, the EA-treated rats performed significantly better at 24 h after ischemia compared with the M group rats, but the difference between the two groups at 2 h was not significant. This finding showed that EA treatment requires time to have an effective role in neurological protection.

### 3.2. EA Treatment Was Neuroprotective against CIRI after Focal Cerebral Ischemia

**Figure 2:**
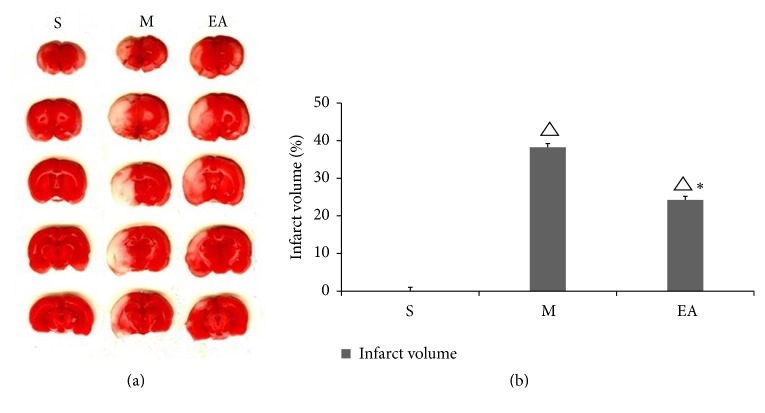
EA treatment reduced the infarct volume. Injury in the brain slices was determined by the TTC method 24 h after MCAO with and without EA treatment. The EA group showed smaller MCAO-evoked infracted areas compared to the M group. For the column, infarct volume was calculated in the three groups (Figure 2(b)). “△” means *P* < 0.05 versus S group, while “*∗*” means *P* < 0.05 versus M group.

The neuroprotective effects of EA stimulation were further analyzed in the focal ischemia model. After being narcotized, the brains were collected and dissected into five consecutive coronal sections (2 mm thick). The infracted tissue was visualized by TTC staining with white color in the infarct area and red color in the normal area (Figure 2(a)). EA stimulation at GV20 and ST36 significantly decreased the volume of the ischemic area (group EA versus group M). Statistically significant differences were discerned between groups S and M, S and EA, and M and EA (Figure 2(b)).

### 3.3. EA Treatment Upregulated the Protein Expression Levels of EPO and EpoR

**Figure 3:**
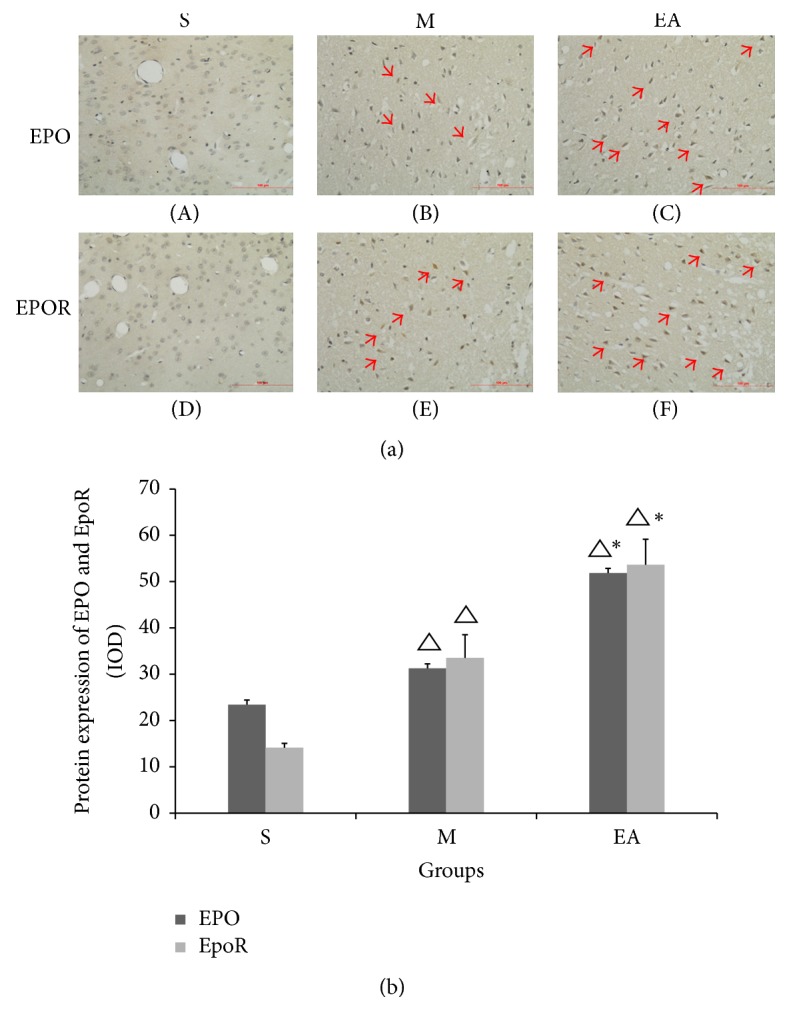
Protein quantities of EPO and EpoR at 24 h in rats undergoing MCAO injury. Group EA (red arrows) showed strong EPO and EpoR immunoreactivity in the cell bodies and a lower integrated optical density was observed in group M. The immunohistochemical expression in group S was the lowest compared with group M and group EA (Figure 3(a)). For the column, the integrated optical density was calculated in three random images of every slice (Figure 3(b)). ^△^*P* < 0.05 versus S group, ^*∗*^*P* < 0.05 versus M group. Scale bar = 100 *μ*m.

We confirmed the hypothesis that EA treatment could upregulate the protein quantities of EPO and EpoR in the ischemic penumbra after 24 h of MCAO. In the S group, low EPO and EpoR immunoreactivity was shown in the majority of the cells (Figures 3(A) and 3(D)). The red arrows show that the protein expression levels were higher in the M group (Figures 3(B) and 3(E)) and that noticeable increases in the immunostaining of each protein were observed in the EA group (Figures 3(C) and 3(F)). Statistically significant differences were discerned between groups S and M, S and EA, and M and EA (Figure 3(b)).

### 3.4. EA Treatment Upregulated the Protein Expression Levels of JAK2 and STAT3

**Figure 4:**
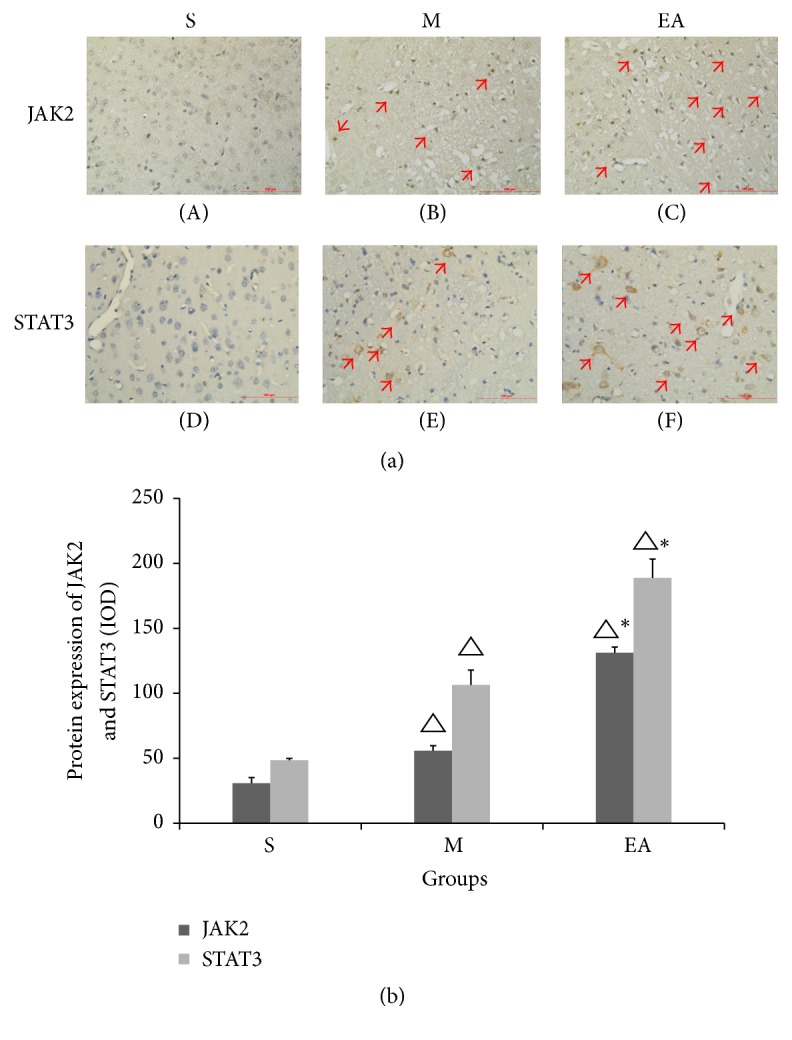
Protein expression levels of JAK2 and STAT3 at 24 h after MCAO (Figure 4(a)). Group EA (red arrows) showed strong JAK2 and STAT3 immunoreactivity in the cell bodies and a lower integrated optical density was observed in group M. The immunohistochemical expression in group S was the lowest compared with group M and group EA. For the column, the integrated optical density was calculated in three random images of every slice (Figure 4(b)). ^△^*P* < 0.05 versus S group, ^*∗*^*P* < 0.05 versus M group. Scale bar = 100 *μ*m.

Next, we confirmed the hypothesis that EA treatment could upregulate the protein expression levels of JAK2 and STAT3. For rats in the S group, low JAK2 and STAT3 immunoreactivity was found in most of the cells (Figures 4(A) and 4(D)). The red arrows show that the protein expression levels were higher in the M group (Figures 4(B) and 4(E)), and noticeable increases in the immunostaining of each protein were observed in the EA group (Figures 4(C) and 4(F)). Statistically significant differences were discerned between groups S and M, S and EA, and M and EA (Figure 4(b)).

### 3.5. Effects of EA Stimulation on MCAO-Induced Neuronal Apoptosis

**Figure 5:**
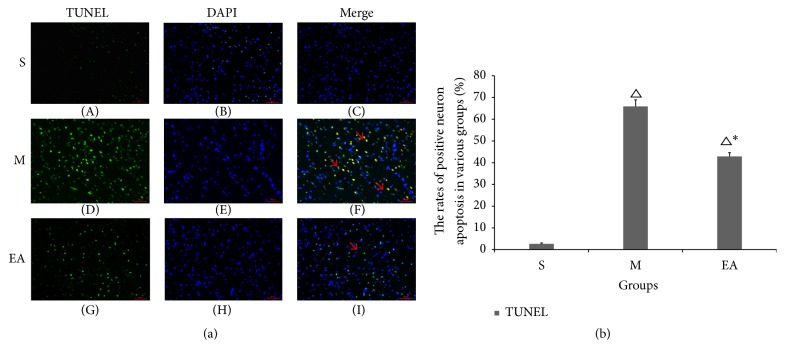
The pictures revealed the immunofluorescence expression of TUNEL-positive cells in the S, M, and EA groups (red arrows). For the column, the rate of positive neuron apoptosis was calculated (Figure 5(b)). ^△^*P* < 0.05 versus S group, ^*∗*^*P* < 0.05 versus M group. Scale bar = 50 *μ*m.

To investigate the influence of electroacupuncture on cell apoptosis in the lesion boundary zone in the three rat groups, TUNEL staining was conducted. The results revealed that rare TUNEL cells were expressed in the S group and that the immunofluorescence expression of nuclear TUNEL-positive cells in the EA group was lower than in group M (Figure 5(a)). Statistically significant differences were observed between the three groups (Figure 5(b)).

### 3.6. Colocalization of JAK2 with Cellular Markers (NeuN and GFAP) and Double-Labeling Immunofluorescent Staining of TUNEL with EPO

**Figure 6:**
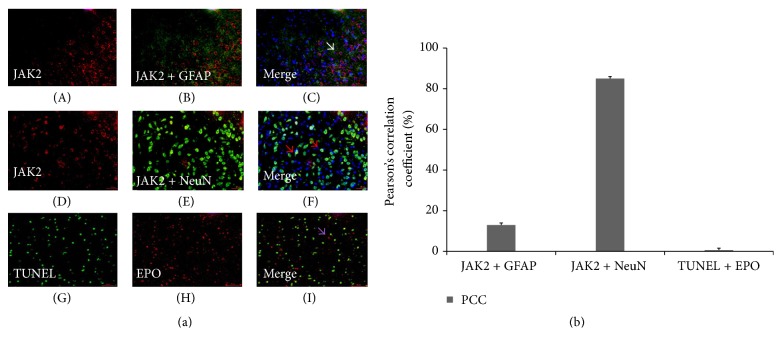
Double-labeling immunofluorescent localization of JAK2 and cellular markers (NeuN and GFAP) 24 h after CIRI in the M group is shown in Figure 6. The colocalization of TUNEL and EPO is also shown. Many neurons in the M group colocalized with JAK2 in Figures 6(D)–6(F) (red arrows), and JAK2 immunofluorescence was most often dissociated from GFAP immunofluorescence in Figures 6(A)–6(C) (white arrows). Purple arrows showed nearly complete dissociation of EPO and TUNEL-positive cells (Figures 6(G)–6(I)). Scale bar = 50 *μ*m. The calculation of PCC for JAK2 and GFAP was less than 20%, while the value for JAK2 and NeuN was more than 80%. The observation of dissociation between TUNEL-positive cells and EPO-labeled cells was supported by the results of coefficients calculation: PPC was less than 10%.

To evaluate the neuronal and astrocytic colocalization of JAK2 in the ischemic penumbra of CIRI rats, double-labeling immunofluorescent staining of JAK2 with NeuN and JAK2 with GFAP is shown in Figure 6. Few astrocytes in the M group colocalized with JAK2, and most JAK2-positive cells colocalized with NeuN (Figures 6(A)–6(F)). Double-labeling immunofluorescent staining of TUNEL with EPO showed that the TUNEL-positive cells and the EPO-labeled cells rarely displayed strong colocalization (Figures 6(G)–6(I)). PCC values for the colocalization of JAK2 with GFAP, JAK2 with NeuN, and TUNEL-positive cells with EPO-labeled cells were shown in Figure 6(b).

### 3.7. Results of the mRNA Expressions of EPO, EpoR, JAK2, and STAT3 Further Confirmed the Results of the Protein Expression Evaluation

**Figure 7:**
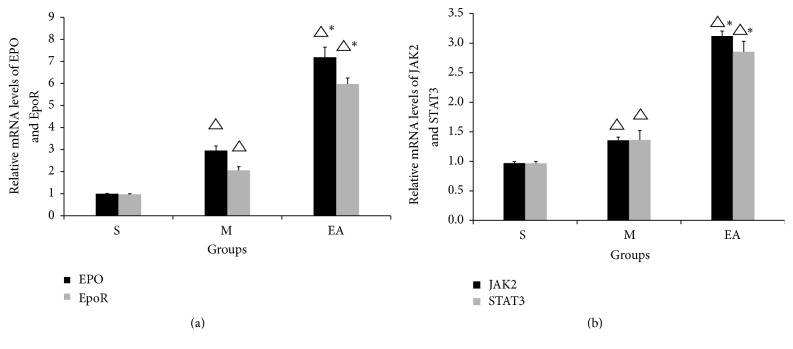
EA treatment regulated the transcriptional levels of EPO, EpoR, JAK2, and STAT3. Twenty-four hours after CIRI, the penumbra area of the rat brain was isolated for total RNA extraction. In Figure 7(a), quantification of the qPCR data for EPO and EpoR mRNA in group S, group M, and group EA. In Figure 7(b), the expression levels of JAK2 and STAT3 mRNA in the three groups. The data are shown as the mean ± SEM in three independent experiments. Black columns represent the expression of EPO and JAK2 and the gray columns represent EpoR and STAT3. ^△^*P* < 0.05 versus the S group, ^*∗*^*P* < 0.05 versus the M group.

The influence of EA treatment on the mRNA expressions of EPO, EpoR, JAK2, and STAT3 was applied by qPCR in the penumbra area of the CIRI rats. Four proteins showed similar regulatory trends. Low-level expression of all EPO, EpoR, JAK2, and STAT3 mRNA was revealed in group S (Figure 7). The results also showed that the mRNA expression of EPO, EpoR, JAK2, and STAT3 dramatically increased in group EA compared with the M group (*P* < 0.05). Our results confirm the histopathological and immunostaining results that the EPO-mediated cellular protective function was preceded by EA treatment.

### 3.8. Western Blot Analysis

#### 3.8.1. EA Treatment Induced Upregulated EPO and EpoR Activity in the Ischemic Penumbra

**Figure 8:**
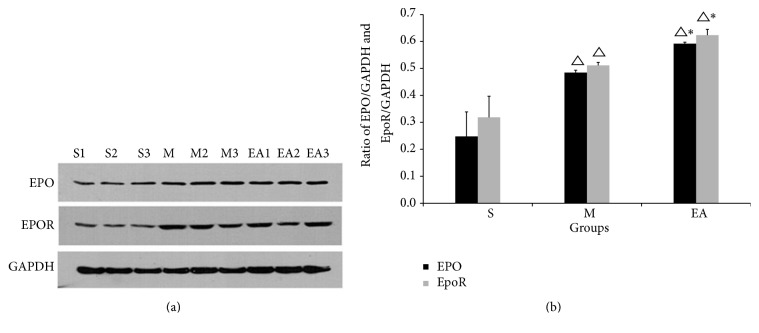
Effect of EA treatment on the expression of EPO and the EpoR. The Western blotting analysis of EPO and EpoR protein levels are shown in the pictures. Black columns represent the expression of EPO and the gray columns represent the EpoR. ^△^*P* < 0.05 versus the S group, ^*∗*^*P* < 0.05 versus the M group.

To clarify the activation of EPO and the EpoR in the EA-mediated protective effect from CIRI, a Western blot analysis was employed to explore the expression levels of EPO and the EpoR (Figure 8(a)). The two proteins showed similar regulatory trends. Lower expression levels of EPO and EpoR proteins were observed in S group. The expression levels significantly increased with the induction of CIRI in the M group. The increased EPO and EpoR activities in CIRI rats were activated by EA treatment in the EA group (Figure 8(b)).

#### 3.8.2. EA Treatment Regulated the Expression and Phosphorylation Levels of JAK2/STAT3 in Rats 24 h after CIRI

**Figure 9:**
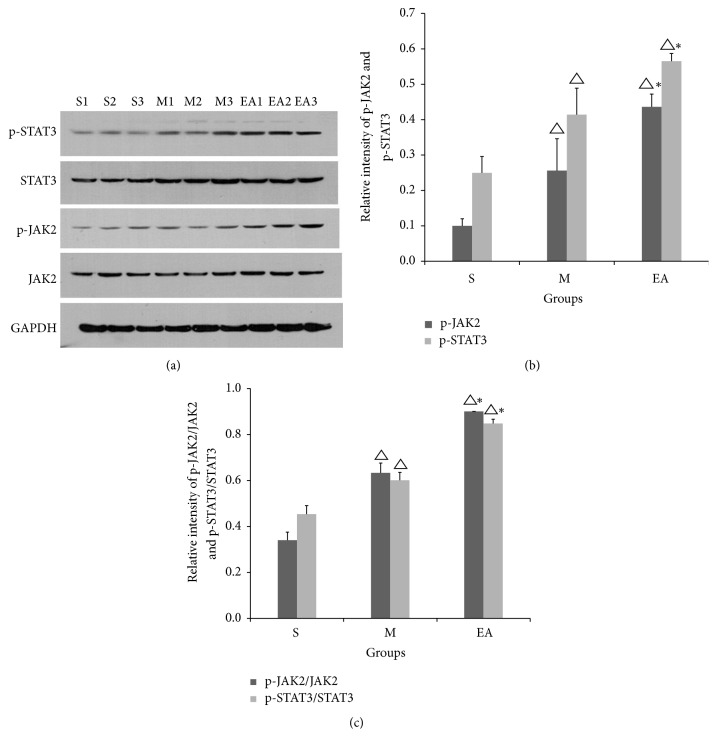
Effect of EA stimulation on the expressions of p-JAK2 and p-STAT3 (Figures 9(a) and 9(b)). The ratios of p-JAK2/JAK2 and p-STAT3/STAT3 are also revealed in the table (Figure 9(c)). The data are expressed as the mean ± SEM. ^△^*P* < 0.05 compared to the S group, ^*∗*^*P* < 0.05 compared to the MCAO group.

Since EPO has been reported to activate JAK2 and its downstream STAT3 in the ischemic penumbra area, the relative mRNA levels of p-JAK2, JAK2, p-STAT3, and STAT3 are shown in Figure 9. In addition to the upregulation of EPO and the EpoR, the protein levels of p-JAK2 and p-STAT3 increased 24 h after CIRI in rats that received EA treatment, as shown in the EA group.

## 4. Discussion

Stroke can be classified as one of the common causes of mortality and disability around the world. Since ancient times, acupuncture has been commonly used to restore the internal balance and harmony of the body through the insertion of several needles or sometimes lasers into defined points using traditional Chinese medicine (TCM) theories. A considerable number of studies that evaluate the effectiveness of acupuncture intervention have been conducted for many diseases, such as migraine attacks [21], traumatic brain injury [22], acute cerebral ischemia/reperfusion injury [23], Alzheimer's disease [24], and depression [25].

According to Institutes of Health consensus panel [26], acupuncture is an important complementary and alternative treatment with the advantages of simple and convenient operation. Both acupuncture and EA treatment have been widely applied for the treatment of stroke complications, including hemiplegia, aphasia, and extremity numbness. EA treatment, which delivers electrical stimulation to the acupoints with an acupunctoscope device, has been increasingly recommended in modern times. The two acupoints Baihui (GV20) and Zusanli (ST36), which are based on TCM theory, were selected for the research. The Baihui acupoint (GV20) is located at the middle of the vertex, on the line that connects the apexes of the two ears and is a commonly used acupoint for the relief of headache, depression, dizziness due to vascular dysfunction, and endocrine, immune, and/or nervous system symptoms. GV20 was identified to be involved in the treatment of cerebral ischemic injury by improving motor function, balance function, and the activities of daily living in experimental animals with stroke injuries. GV20 was also reported to exert neuronal protective effects by inhibiting apoptosis-related protein expression in rats with cerebral ischemia [27]. The location of ST36 is on dermatome L5 and is located lateral from the anterior crest of the tibia and 3 cun (the breadth of the four fingers of the patient's hand close together at the proximal interphalangeal joint of the middle finger) below the knee laterally. Accordingly, EA stimulation of acupoint ST36 was determined to be effective for the production of a protective effect in brain ischemia patients. Previous studies reported that acupuncture at ST36 induced the modulation of inflammatory responses in internal organs [28], increased enkephalin in the brain [29], regulated autoimmune disorders of the central nervous system, and so on [30]. Further experimental studies in animals have revealed that needling at the ST36 acupoint can inhibit cell apoptosis [31]. Our results that show improved neuronal function, decreased TUNEL-positive cells, and a reduced volume of the ischemic area demonstrate that EA treatment at GV20 and ST36 was beneficial for the CIRI rats.

EPO, a hormone produced by many tissues such as the kidney and the liver, acts via the EpoR to exert a strong erythropoietic effect. The function of the EPO/EpoR pathway in nonhematopoietic tissues has recently been revealed. The activation of the EPO/EpoR pathway has recently been demonstrated in several nonhematopoietic tissues, such as the brain, lung, and breast [32–34]. Accordingly, the expression of EPO and the EpoR has also been detected in the central nervous system. Extensive research has shown that the hypoxia-induced rescue proteins EPO and EpoR can sustain activation to exert neuroprotective effects. RhEpo therapy and nonhematopoietic EpoR agonist drugs have been investigated and further developed to protect neuronal cells by preventing apoptosis in animal models [35, 36]. However, the clinical application of these drugs warrants further research in ongoing clinical trials. Our finding of high EPO and EpoR mRNA levels in the brain and our demonstration of the hypoxic upregulation of both of these proteins are suggestive of the mechanisms through which the EA therapeutic modality at GV20 and ST36 may contribute to ischemic stroke.

The EPO/EpoR axis was reported to be activated through an interaction with intracellular tyrosine kinases JAK2. JAK2, in turn, phosphorylates STAT3, and STAT3 subsequently dimerizes and accumulates in the nucleus where it binds to target genes with specific sites to elicit mitogenic signals. The physiological and pathological processes of inflammation, cell survival, proliferation, and cell angiogenesis were regulated by JAK2/STAT3 signaling after CIRI in animal models [37, 38]. Several downstream target gene products, such as Bcl-2, Bcl-XL, and the Caspase and Bax protein families, were activated after JAK2/STAT3 binding to specific gene promoters [39, 40]. Studies have shown that the activation of the JAK2/STAT3 pathway after experimental stroke could improve functional performance and/or decrease cell apoptosis [41, 42]. Since both the EPO/EpoR and the JAK2/STAT3 pathways have been implicated as molecular targets for ischemic stroke prevention and treatment, the activation of both of these cell pathways and their downstream signaling cascades is recognized as a promising therapeutic approach for stroke patients.

In our study, EA stimulation at acupoints GV20 and ST36 was shown to serve as a therapeutic strategy for the attenuation of neurofunction deficits, reductions in the cerebral infarction area, and enhancement of the protein and mRNA expression of EPO, the EpoR, p-JAK2, JAK2, p-STAT3, and STAT3 in the model of CIRI. Our results were consistent with our hypothesis that the activated EPO/EpoR- JAK2/STAT3 signaling pathway could be induced by EA stimulation at acupoints GV20 and ST36.

Apoptosis is responsible for an important portion of CIRI. The TUNEL staining in the ischemic penumbra area of EA group rat brains indicated that treatment with EA was capable of decreasing apoptotic cells compared to M group rats (Figure 5). The dissociation between EPO-positive and TUNEL-labeled apoptotic cells (Figure 6) further indicated that the inhibition of cell apoptosis was the possible mechanism of the EA-mediated protective effects against CIRI. The results indicated the possibility that, in the areas of impending apoptotic neuronal injury, neighboring cells may attempt to increase EPO-signaling so that the injury can be neutralized by the neuroprotection of EPO. However, in the surrounding areas of an ischemic infarct at the site of very severe injury, the nearly complete dissociation of EPO and TUNEL-positive cells could indicate a lack of the EPO cell pathway. Our results confirm previous observations in humans [43].

In conclusion, EA treatment represents complementary and alternative strategies for the treatment of brain ischemia. In this study, we observe that the expression levels of EPO/EpoR and JAK2/STAT3 dramatically increased and that EA stimulation at GV20 and ST36 may induce EPO/EpoR-mediated cells apoptosis via the JAK2/STAT3 pathway. However, the relationships among EA treatment, JAK2/STAT3 signal transduction and apoptosis pathways, and the exact mechanism of acupuncture stimulation-signal conditioning need to be further studied.
